# Epidemiologic Trends in Maxillofacial Trauma Surgery in Germany—Insights from the National DRG Database 2005–2022

**DOI:** 10.3390/jcm13154438

**Published:** 2024-07-29

**Authors:** Axel Meisgeier, Simon Pienkohs, Florian Dürrschnabel, Laura Moosdorf, Andreas Neff

**Affiliations:** 1 Department of Oral and Craniomaxillofacial Surgery, UKGM GmbH, University Hospital Marburg and Faculty of Medicine, Philipps University, 35043 Marburg, Germany; simonpatrik.pienkohs@uk-gm.de (S.P.); duerrsch@med.uni-marburg.de (F.D.); neffa@med.uni-marburg.de (A.N.); 2 Center for Orthopaedics and Trauma Surgery, UKGM GmbH, University Hospital Marburg and Faculty of Medicine, Philipps University, 35043 Marburg, Germany; lmoosdor@med.uni-marburg.de

**Keywords:** fracture epidemiology, geriatrics, maxillofacial fracture, traumatology, time trends

## Abstract

**Background:** Maxillofacial trauma (MFT) caused by falls, interpersonal violence or traffic accidents leading to fractures of different facial regions, including the midface and the mandible, are common clinical conditions requiring open reduction and internal fixation. The aim of this study was to analyze the incidence and time trends in MFT-associated surgeries regarding different facial regions in the German healthcare system over time. **Materials and methods:** Nationwide data regarding the national diagnosis-related group (DRG) inpatient billing system was received from the German Federal Statistical Office for the years 2005–2022. We estimated the age–gender standardized incidence of MFT-associated procedures classified by the Operation and Procedure Classification System (OPS) and evaluated age- and gender-adjusted time trends using Poisson regression analysis. **Results:** The total standardized incidence rate of MFT-associated procedures in the observational period 2005–2022 was 25.1 (♀13.3; ♂37.5) per 100,000 person-years within a slight significant annual decrease of 0.5%. A significant increase in the incidence of MFT-related procedures within the observational period was found in older adults from 60 to 79 years (+55.1%; ♀+54.8%; ♂+56.3%) and elderly patients over 80 years (+66.7%; ♀+59.1%; ♂+85.1%). Other significant trends are decreases in MFT-related procedures performed in children from 0–14 years (−28.1%; ♀−30.3%; ♂−27.3%) and young adults between 15 and 35 years (−20.4%; ♀−7.3%; ♂−22.5%). **Conclusions:** MFT-associated surgery is a persisting challenge in the German healthcare system. There is an ongoing transition in MFT-associated surgeries from younger to older patients beyond the scope of demographic change, highlighting the increasing importance of interdisciplinary treatment of patients with pre-existing conditions in maxillofacial surgery. Implementation of injury prevention measures might be beneficial in this population.

## 1. Introduction

Maxillofacial fractures represent a significant category of bone fractures, with a substantial impact on healthcare, quality of life and a considerable economic burden [1,2,3,4]. Maxillofacial fractures encompass a range of injuries affecting the mandible, the maxilla, the zygomatic complex, the orbital walls, the teeth, and the paranasal and frontal sinuses. A significant proportion of patients in maxillofacial departments in Germany are those with maxillofacial trauma (MFT) [1]. It is notable that traumatic facial injuries are particularly prevalent among young adult males [5,6]. The most common causes are physical violence, road traffic accidents, falls and sports and leisure injuries. Such occurrences are subject to pronounced seasonal trends and fluctuations [1,7,8]. The standard procedure for fractures of most facial regions is open reduction and internal fixation [9,10]. Despite the growing evidence supporting the superiority of surgical treatment for mandibular condylar process fractures over the past two decades, conservative treatment with closed reduction and maxillomandibular fixation remains a viable option [11]. Virtual image segmentation, CAD/CAM planning, navigated surgery, additively manufactured patient-specific implants, and intraoperative three-dimensional imaging have significantly expanded the possibilities of surgical treatments in all areas of facial traumatology by personalizing surgical therapy. This may lead to improvements in the predictability of surgical methods and improved functional and aesthetic outcomes, as well as an extension of the range of indications for open surgical versus conservative treatment options [12].

Historically, in the era of early industrialization, the incidence of maxillofacial fractures increased due to occupational accidents, leading to improvements in occupational safety. With the development of individual mobility, maxillofacial fractures caused by traffic accidents increased significantly until the introduction of airbags and mandatory seat belts in cars. Conversely, sports and leisure accidents, as well as fractures caused by physical violence, have increased in recent decades [13,14]. There is only scarce data on the incidence and time trends of maxillofacial fractures in Germany in recent years. Data from a regional single-center study suggest a slight overall increase in midfacial fractures, with a strong increase among elderly people and a slight decrease in midfacial fractures among younger people [6]. Nevertheless, there is a paucity of population-based data concerning trends in the incidence of maxillofacial fractures. Studies from other industrialized countries remain indistinctive. Some studies suggest an increasing incidence of MFT-associated fractures [15,16,17]. Others show a decrease in the number of facial fractures [18,19,20]. To the best of our knowledge, there is no nationwide investigation representing a Central European country like Germany. The objective of this study was, therefore, to estimate the national and regional age- and gender-standardized incidence of MFT-associated procedures in Germany between 2005 and 2022 and evaluate age- and gender-adjusted time trends.

## 2. Materials and Methods

The national diagnosis-related groups (DRG) inpatient billing system encompasses data from all German hospitals that use the DRG system. The DRG system covers more than 99% of inpatient treatments in Germany. Hospitals are legally obliged to furnish comprehensive data concerning the care provided, including patient demographics, diagnoses, comorbidities, complications, and procedures. Surgical procedures performed between 2005 and 2022 were classified according to the OPS (Operation and Procedure Classification System), a German modification of the ICPM (International Classification of Medical Procedures). All diagnoses were coded in accordance with the ICD-10GM (German version of the International Classification of Diseases and Related Health Problems, 10th Edition). A comprehensive account of all surgical procedures pertaining to open reduction and internal fixation, classified according to the OPS (coded 5-760, 5-761, 5-762, 5-763, 5-764, 5-765, 5-766 and 5-767) were provided by the German Federal Statistical Office (Statistisches Bundesamt—Destatis. Genesis-Online. Data license by-2-0). The OPS classification of the surgical procedures distinguishes between 5-760 fractures of the lateral midface, including zygomatic bone and zygomatic arch fractures, 5-761 fractures of the central midface, including fractures at Le Fort I and Le Fort II levels, 5-762 fractures of the central and lateral upper midface including fractures at Le Fort III Level and nasoorbitoethmoidal fractures, 5-763 combined and complex midfacial fractures, 5-764 mandibular body fractures, 5-765 fractures of the mandibular ramus and the condylar process, 5-766 orbital wall fractures and 5-767 frontal sinus fractures. The dataset comprises all inpatient procedures pertaining to maxillofacial fractures conducted in Germany over the specified observational period, classified by year, gender, age, and federal state. The number of MFT-associated procedures per year (PPY) was calculated and reported. The mean age of patients was calculated. The normality of the distribution of continuous variables was evaluated using the Kolmogorov–Smirnov test. Continuous variables with a normal distribution were presented as the mean and standard deviation. The means of two continuous, normally distributed variables were compared using an independent sample Student’s *t*-test. A *p*-value of less than 0.05 was considered to indicate a statistically significant difference. Furthermore, population-adjusted rates of MFT-associated procedures per 100,000 person-years were calculated using population data provided by the German Federal Statistical Office and reported with a 95% confidence interval. To eliminate the potential impact of seasonal variations, the analysis of MFT-associated procedures was conducted on an annual basis. 

The primary analysis was conducted on the entire population, with subsequent analyses stratified by gender, age group, affected facial region, and federal state. We calculated the crude and age–sex standardized incidence rates of MFT-associated procedures for each calendar year using the German population from the latest census (2011) as the standard population. To exclude bias due to demographic heterogeneity over time, we divided the population into five age classes: 0–14, 15–34, 35–59, 60–79, and ≥80 years. To ascertain male and female incidence rates, age standardization was performed with the aforementioned age classes using the age distribution of the male and female standard population, resulting in different weights for both genders. The 95% confidence intervals (95% CI) of the crude and standardized incidences were calculated using the delta method. Separate Poisson regression models were constructed to investigate time trends, fitted with incidence rates of MFT-associated procedures as dependent variables and age group, gender, facial region, federal state and year starting from baseline year 2005 as independent variables. The age group of 35–59 years and the female sex were used as a reference group. All models were adapted through a descale adjustment to account for the overdispersion of the outcome variable. The statistical analysis was performed using IBM SPSS Statistics Version 29.0 (IBM Deutschland GmbH, Böblingen, Germany).

## 3. Results

**Figure 1:**
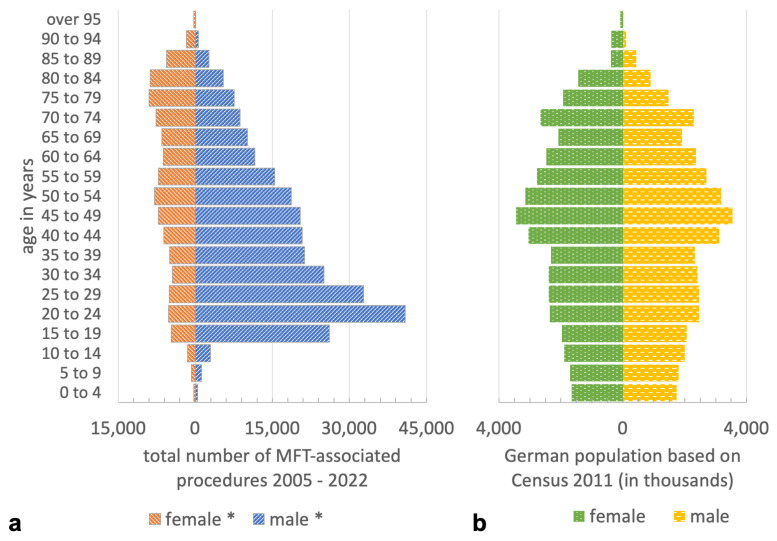
(**a**) Age–gender diagram of the total number of MFT-associated procedures 2005–2022. (**b**) Age–gender diagram of the German population based on census 2011.

A total of 374,143 MFT-associated procedures were registered in Germany between 2005 and 2022 and were thus included in the study. The distribution of age, gender, facial region, and year of treatment for MFT is presented in Figure 1 and Table 1.

**Figure 2:**
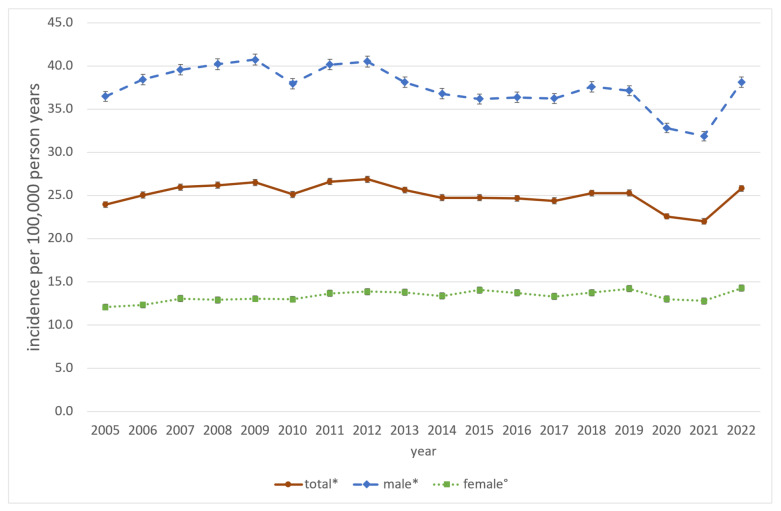
Standardized overall and gender-specific incidence of MFT-associated procedures, Germany, 2005–2022. * Significant decrease (*p*-value time trend Poisson Regression model < 0.05); ° Significant increase (*p*-value time trend Poisson Regression model < 0.05).

**Figure 3:**
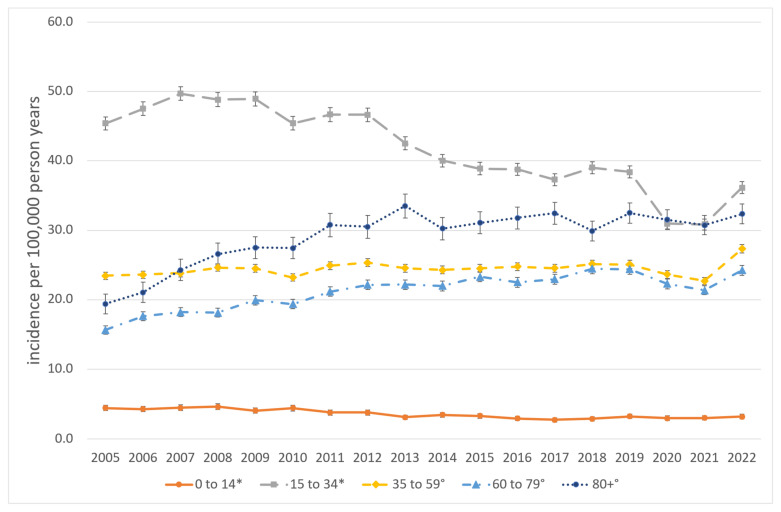
Standardized age-specific incidence of MFT-associated procedures, Germany, 2005–2022. * Significant decrease (*p*-value time trend Poisson Regression model < 0.05); ° Significant increase (*p*-value time trend Poisson Regression model < 0.05).

**Figure 4:**
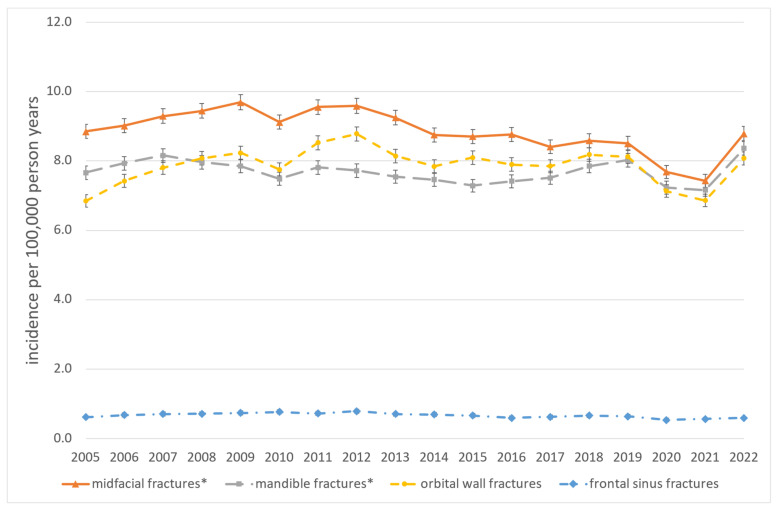
Standardized procedure-specific incidence of MFT-associated procedures, Germany, 2005–2022. * Significant decrease (*p*-value time trend Poisson Regression model < 0.05); ° Significant increase (*p*-value time trend Poisson Regression model < 0.05).

The majority of procedures were performed on patients aged 35 years or older (59.5%) and male (72.9%). The mean age exhibited an increase over the course of the study period, from 41.6 years between 2005 and 2013 to 46.3 years between 2014 and 2022 (*p* < 0.01). The most common fractures necessitating surgical intervention were orbital wall fractures (n = 117,666; 31.5%) followed by lateral midfacial fractures (viz. fractures of the zygomatic complex) (n = 102,491; 27.4%) and mandibular body fractures (n = 66,717; 17.8%). The mean age was highest for combined and complex midfacial fractures at 49.0 years and the lowest for mandibular body fractures at 37.3 years. The age- and gender-standardized incidence rates of procedures associated with MFT are presented in Table 2 and Table 3 and Figure 2, Figure 3 and Figure 4 for the total population and stratified by gender, age and procedure. 

The overall standardized incidence rate of MFT-associated procedures during the observational period 2005 to 2022 was 25.1 per 100,000 person-years [95% CI: 25.0–25.2], with a range from 22.0 [21.7–22.3] in 2021 to 26.9 [26.5–27.2] in 2012. There was a slight shift in the gender distribution from 75.1% in males and 24.9% in females in 2005 to 70.7% and 29.3% in 2022, respectively. In males, the overall incidence was 37.5 [37.4–37.7], spreading from 31.9 [31.3–32.4] in 2005 to 40.8 [40.1–41.4] in 2009. In females, the total incidence rate was 13.3 [13.3–13.4], varying between 12.1 [11.7–12.4] in 2005 and 14.3 [13.9–14.6] in 2022. Among the different age groups, children from 0 to 14 years had the lowest incidence rates, with a total of 3.6 [3.5–3.7] ranging from 2.7 [2.4–3.0] in 2017 to 4.6 [4.2–4.9] in 2008. An incidence of 4.4 [4.0–4.7] in 2005 and 3.2 [2.9–3.5] in 2022 represents a strong decrease of −28.1% in procedures associated with pediatric fractures during the observational period. Adolescents and young adults from 15 to 34 years had the highest total incidence rate, with 36.2 [35.3–37.0] differing between 49.7 [48.7–50.7] in 2007 to 30.8 [30.0–31.6] in 2021. In 2022, the incidence was 36.2 [35.3–37.0], a decrease of −20.4% within the observational period. In middle-aged adults from 35 to 59 years, the overall incidence was 24.4 [24.3–24.6], spreading from 22.7 [22.1–23.2] in 2021 to 27.3 [26.6–27.9] in 2022.

Older adults from 60 to 79 years showed an incidence rate of 21.2 (21.0–21.4), varying from 15.6 [15.0–16.2] in 2005 to 24.5 [23.7–25.2] in 2018. In 2022, the incidence was 24.2 [23.5–24.9], an increase of 55.1% within the observational period. Among elderly people (80 years and older). The total incidence rate was 29.1 [28.7–29.5], ranging from 19.4 [18.0–20.8] in 2005 to 32.5 [31.0–34.0] in 2019. With an incidence of 32.3 [30.9–33.8] in 2022, this represents the strongest increase with 66.7% during the observational period.

**Figure 5:**
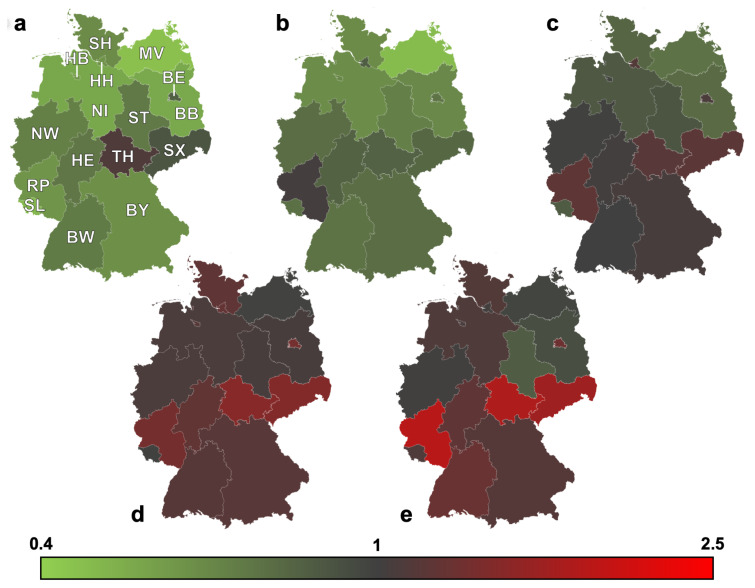
Relative risk for MFT-associated procedures Germany 2014–2022 vs. 2005–2013 stratified by age-group and federal state. (**a**) Age group 0–14 years. (**b**) Age group 15–34 years. (**c**) Age group 35–59 years. (**d**) Age group 60–79 years. (**e**) Age group over 80 years. * *p*-value < f 0.05; SH—Schleswig Holstein; MV—Mecklenburg-Vorpommern; HH—Hamburg; HB—Bremen; NI—Lower saxony; BE—Berlin; BB—Brandenburg; ST—Saxony-Anhalt; NW—North Rhine-Westphalia; HE—Hesse; TH—Thuringia; SX—Saxony; RP—Rhineland-Palatinate; SL—Saarland; BW—Baden-Wurttemberg; BY—Bavaria.

Reviewing the 16 German federal states individually, the overall incidence of surgical procedures associated with MFT within the observational period ranged from 20.3 [20.0–20.6] per 100,000 person-years in Rhineland-Palatinate to 57.5 [56.1–58.9] in Bremen as shown in Table 4. A single-year minimum was seen in Rhineland-Palatinate in 2006 with 14.7 [13.5–15.9]. A single-year maximum was registered in Bremen with 70.4 [64.0–76.8] in 2008. The results of the time trend of incidence from the relative risk estimation and the fully adjusted Poisson models are shown in Table 5 and Table 6 and Figure 5. We observed only a slight overall decrease in the incidence of MFT-associated procedures with a weak but significant annual percentage decrease of 0.5% in the observational period (relative risk per calendar year 0.995; 95% CI: 0.994–0.996, *p* < 0.05). The decrease was stronger in males (−0.9% per year, RR: 0.991; 0.990–0.992; *p* < 0.05), while in females, there was a slight increase (+0.7% per year, RR:1.007; 1.005–1.008; *p* < 0.05). Significant decreases were observed in age groups 0–14 years (−2.5% per year, RR: 0.975; 0.970–0.979; *p* < 0.05) and 15–34 years (−2.4% per year, RR: 0.976; 0.975–0.977; *p* < 0.05). In contrast, age groups over 80 years (+1.9% per year, RR: 1.019; 1.016–1.021; *p* < 0.05) and 60–79 years (+1.9% per year, RR: 1.019; 1.017–1.020; *p* < 0.05) showed a significant increase. Only a small change was seen in adults from 35 to 59 years (+0.2% per year, RR: 1.002; 1.001–1.003; *p* < 0.05). Slight but significant decreases were observed for midfacial fractures (−1.0% per year, RR: 0.990; 0.989–0.991; *p* < 0.05) and mandibula fractures (−0.4% per year, RR: 0.996; 0.995–0.998; *p* < 0.05).

No significant trends were seen in orbital wall fractures and frontal sinus fractures. A significant increase of MFT-associated procedures was found in 5 of 16 federal states, ranging from +0.3% per year in Hamburg (RR: 1.003; 1.001–1.005; *p* < 0.05) to +1.3% per year in Saxony (RR: 1.013; 1.010–1.015; *p* < 0.05). A significant decrease in MFT-associated procedures was found in 10 of 16 federal states, ranging from −0.3% per year in Bavaria (RR: 0.997; 0.995–0.999; *p* < 0.05) to −3.4% per year in Mecklenburg-Vorpommern (RR: 0.966; 0.964–0.969; *p* < 0.05). 

## 4. Discussion

The field of traumatology represents a persistently evolving challenge across numerous medical specialties. Therefore, MFT remains a primary area of interest in oral and maxillofacial surgery. As the most common causes of MFT, such as physical violence, traffic accidents, falls, sports and leisure injuries, remain prevalent, MFT remains an important cause of hospitalization in oral and maxillofacial surgery departments [1]. 

Nevertheless, reliable population-based data concerning long-term trends, age and gender distributions in the incidence of MFT-associated procedures are scarce. They may be inconclusive due to the diverse clinical presentations and therapeutic management options. The majority of available data were gathered from single-center case series, which may not be representative of the broader population. To the best of our knowledge, this is the first population-based study to examine the incidence of MFT-associated procedures utilizing Germany’s DRG inpatient billing database, which encompasses data on the entire German population. Within the observational period from 2005 to 2022, we found a mean incidence of MFT-associated procedures with open reduction and internal fixation of 25.1/100,000 person-years in Germany. As in our case with a gender ratio of 2.8:1, many previous studies have shown that men (37.5/100,00 person-years) are a high-risk group for facial fractures compared to women (13.3/100,000 person-years) [6,17,20,21,22,23,24,25,26,27]. One reason for this could be the fact that men tend to be more risk-taking, which can have a negative impact on social confrontations, road traffic, occupational activities or sports [27,28]. We found a slight but significant decrease in MFT-associated procedures among males with an annual percentage decrease of 0.9% while the incidence in women is slightly increasing at 0.7% per year, resulting in a decreasing gender ratio from 3.0:1 in the first half of the observational period to 2.6:1 in the second half of the observational period. Recent literature supports this trend towards a more equal male-to-female ratio in the last decades [23,27,29]. 

Overall, we found a slight but significant decrease (−0.5% per year) in the incidence of MFT-associated procedures within the observational period. This is in accordance with population-based data from other industrialized countries showing a slight decrease in the incidence of facial fractures in countries with high socio-demographic index (SDI) between 1990 and 2019 [3,25]. Several single-center studies from developed countries also indicate a mild decrease in maxillofacial trauma incidence in recent decades [20,22,30,31]. However, some studies also describe an increasing incidence in individual centers or regional evaluations [6,17,23]. To resolve this apparent contradiction, it is worth looking at the various subgroups with their different underlying effects that run in parallel. First, our study clearly depends on age. Pediatric patients aged 0–14 years (−2.5% per year), as well as adolescents and young adults aged 15–34 years (−2.4% per year), show a strong significant decrease in the incidence of MFT-associated procedures, while adults from 35–59 years (+0.2% per year) show no relevant changes. Older adults from 60–79 years (+1.9% per year), as well as elderly people over 80 years (+1.9% per year), show a strong significant increase in the incidence of MFT-associated procedures. 

The phenomenon of an age-dependent change in the incidence of MFT has societal-wide implications and is not confined to specific regions or genders. However, just as there are marked variations in fracture incidence between different international countries, there are also marked regional differences in the incidence and temporal trends of MFT-associated procedures at the national level. The higher incidences in densely populated city-states such as Hamburg, Bremen and Berlin are noticeable, as are the sharp declines in sparsely populated regions such as Mecklenburg-Vorpommern and Brandenburg. The reasons for this are most likely to be found in the different social and infrastructural conditions and might be a consequence of the various etiologic factors that change over time, especially with regard to the main causes of maxillofacial trauma, namely interpersonal violence, traffic accidents and falls, which are also influenced by geographic location, population density, economic status, and cultural differences [1,26,28,32,33].

Martinez et al. described a relative decrease in facial fracture patients aged 16–40 years along with a relative increase of patients over 66 years in a US single-center two-cohort study comparing cohorts from 1984–1990 to 2004–2010 in association with a reduction in interpersonal violence and road traffic accidents as well as an increase in falls [24]. Interpersonal violence has been identified as the primary cause of both mandibular and midfacial fractures, particularly among young men in industrialized countries with liberal alcohol legislation [1,26]. In our data, the sharp decline in the incidence of MFT-associated procedures in young male adolescents and adults between 15 and 34 years, with their high baseline incidence, represents the most declining subgroup for MFT-associated procedures in absolute numbers. 

This may indicate that during the observational period from 2005 to 2022, the reduction of interpersonal violence was one of the main reasons for the reduced incidence of maxillofacial fractures. This hypothesis is also supported by the lack of a decline of MFT-associated procedures in women and the rather moderate decline of MFT-associated procedures in women of the same age, for whom physical violence is not the most prominent cause of facial fractures and the shift to a more balanced gender ratio [1,26]. Matching this, Kraft et al. showed a reduction in interpersonal violence associated facial fractures and injuries in the recent decades in a regional study in Austria [34]. As alcohol consumption is known to be a main risk factor for interpersonal violence-associated facial fractures, the overall reduction of alcohol consumption in Germany within the observational period might be beneficial [1,35]. However, there are several studies describing a higher proportion of interpersonal violence in the incidence of facial injuries in different industrialized European and non-European countries, highlighting the strong cultural and regional influences and differences [17,36,37,38,39]. Road traffic accidents are the second most common cause of facial fractures. The decline in road traffic accidents involving personal injury as a result of the almost complete implementation of airbags and safety belts in recent decades to a historic minimum is a second mechanism explaining a decline in facial fractures, especially among younger persons in Germany [40]. The use of protective clothing and helmets in road traffic reached an all-time high during our observational period [41]. This conclusion regarding the influence of road traffic accidents is a common observation across the literature and supported by data from a lot of European and non-European countries over the last decades [17,36,37,38,39]. In elderly people, falls are the most common cause of facial fractures, which are insignificant in other age groups [26]. The increase in fall-associated facial trauma is a known effect from different studies in several countries with an aging society [15,31,42,43]. The significant increase in facial fractures in the age group over 60 years and the increase in average age suggest a significant increase in falls that seems to go beyond the effects of demographic change. Possible reasons for this include an increasingly active lifestyle of elderly people despite the progressive development of neuromuscular and cognitive deficits, including balance and gait disorders, medication side effects and cardiovascular risk factors [44,45,46,47]. Another influencing factor may be the trend to smaller family units with an increasing risk of social isolation of elderly people and their lack of social support [48,49]. 

This study is subject to certain strengths and limitations, which are mainly related to the dataset that was available for analysis. Firstly, it should be noted that the specific reimbursed OPS codes from the national DRG system database only represent MFT treated by open reduction and internal fixation in the analyzed years. The dataset does not include MFT treated non-surgically. Secondly, the study encompasses only those MFT cases necessitating hospital admission and resulting in the billing of a DRG. Patients treated as outpatients or utilizing alternative reimbursement schemes were not included. This could lead to certain groups being underrepresented, for example, older people who are more reluctant to undergo surgery due to pre-existing health conditions. Although the evidence for the superiority of surgical treatment in the area of mandibular condylar process fractures has been proven in the last two decades, conservative treatment with closed reduction and maxillomandibular fixation still plays a certain role and might not be fully covered with the described methods [11]. In this context, an increase in surgical interventions for mandibular condylar fractures should not be misinterpreted uncritically as an increase in the underlying fracture frequency. Given that statutory health insurance in Germany offers comprehensive and affordable healthcare coverage to all citizens, and given that MFT is a serious condition that typically necessitates immediate medical attention and hospitalization, it seems reasonable to assume that the vast majority of the German population can be covered and that a comprehensive overview can be provided. The potential of selection bias in MFT determination due to socioeconomic status is mitigated by the population-based approach. The comprehensiveness of our description is constrained by the limitations of the dataset itself, which provides information at an aggregated level and in limited detail, lacking information about the specific cause and severity of the trauma. Consequently, a more advanced analysis, including anamnestic and diagnostic information, was not feasible. Nevertheless, further evaluation of the reasons for the changing incidence of MFT would be of interest. In addition, the DRG inpatient billing database represents an invaluable resource for German researchers engaged in large-scale clinical epidemiology investigations pertaining to procedures and diseases. Furthermore, the utilization of claims data mitigated the potential for recall bias and misclassification bias, which could otherwise arise in studies based on self-reported data. To the best of our knowledge, this study represents the inaugural examination of the time trend of the incidence and epidemiological distribution of MFT-associated procedures at a national level in Germany since the introduction of the DRG system in 2005. The findings may inform an analysis of the influence of population structure and associated factors on the occurrence and treatment of MFT.

The delineation of both beneficial and adverse effects may inform the future identification of risk groups that may profit from social support. To this end, further investigation of the direct influence of social background should be expanded.

## 5. Conclusions

Our study suggests that the incidence rate of MFT-associated procedures in Germany has decreased slightly in recent decades, with two main trends running in parallel. There is a sharp decline in MFT-associated procedures in adolescents and young adults. This is accompanied by a strong increase in MFT-associated procedures in older adults over the age of 60, which is also the fastest-growing age group in an aging society. As these two trends are not limited to specific regions or genders, MFT-associated surgeries are a persistent challenge in countries with comprehensive healthcare, such as Germany. There is a continuous transition from younger to older patients in MFT-associated surgery, exceeding the expectations of demographic change, emphasizing the increasing importance of interdisciplinary treatment of patients with pre-existing conditions in oral and maxillofacial surgery. The implementation of injury prevention strategies, including exercise, active needs-based medication management, regular eye examinations and the adoption of safety precautions in the home environment, coupled with the reduction of potential hazards such as steps or stairs, could prove beneficial for elderly patient groups, especially super octogenarians.

## Figures and Tables

**Table 1:** Total numbers of MFT-associated procedures in Germany 2005–2022.

		Total	Male	Female
All MFT-associated procedures	number of procedures	374,143	272,897	101,246
	mean age (years, SD)	44.0 (20.7)	39.9 (18.7)	54.9 (22.1)
Year				
2005	number of procedures	19,988	15,017	4971
	mean age (years, SD)	39.2 (19.1)	36.0 (16.8)	48.7 (22.2)
2006	number of procedures	20,800	15,723	5077
	mean age (years, SD)	39.8 (19.4)	36.6 (17.2)	49.8 (22.1)
2007	number of procedures	21,503	16,118	5385
	mean age (years, SD)	40.1 (19.9)	36.4 (17.3)	51.2 (22.7)
2008	number of procedures	21,632	16,308	5324
	mean age (years, SD)	40.8 (20.1)	37.2 (17.8)	51.8 (22.5)
2009	number of procedures	21,830	16,447	5383
	mean age (years, SD)	41.6 (20.3)	37.8 (18.0)	53.3 (22.4)
2010	number of procedures	20,694	15,310	5384
	mean age (years, SD)	42.1 (20.6)	38.3 (18.4)	52.9 (22.6)
2011	number of procedures	21,374	15,764	5610
	mean age (years, SD)	43.0 (20.7)	38.9 (18.4)	54.5 (22.4)
2012	number of procedures	21,753	16,023	5730
	mean age (years, SD)	43.5 (20.7)	39.5 (18.5)	54.5 (22.3)
2013	number of procedures	20,896	15,183	5713
	mean age (years, SD)	44.7 (21.0)	40.5 (18.9)	55.9 (22.1)
2014	number of procedures	20,351	14,760	5591
	mean age (years, SD)	44.9 (20.9)	40.6 (18.7)	56.2 (22.1)
2015	number of procedures	20,792	14,870	5922
	mean age (years, SD)	45.5 (21.1)	41.4 (19.1)	55.9 (22.2)
2016	number of procedures	20,755	14,924	5831
	mean age (years, SD)	45.6 (21.1)	41.3 (19.0)	56.8 (22.0)
2017	number of procedures	20,536	14,839	5697
	mean age (years, SD)	46.3 (21.2)	41.9 (19.4)	57.7 (21.6)
2018	number of procedures	21,213	15,299	5914
	mean age (years, SD)	46.1 (21.2)	41.6 (19.3)	57.6 (21.7)
2019	number of procedures	21,240	15,090	6150
	mean age (years, SD)	46.6 (21.5)	42.2 (19.8)	57.4 (21.8)
2020	number of procedures	18,890	13,214	5676
	mean age (years, SD)	48.0 (21.5)	43.7 (20.0)	57.9 (21.6)
2021	number of procedures	18,370	12,782	5588
	mean age (years, SD)	47.8 (21.6)	43.6 (20.2)	57.5 (21.8)
2022	number of procedures	21,526	15,226	6300
	mean age (years, SD)	47.3 (21.2)	43.3 (19.5)	57.0 (21.9)
Age				
0–14 years	number of procedures	7200	4615	2585
15–34 years	number of procedures	144,266	124,840	19,426
35–59 years	number of procedures	130,068	96,650	33,418
60–79 years	number of procedures	67,336	37,984	29,352
over 80 years	number of procedures	25,273	8808	16,465
Facial region		Total	Male	Female
fracture of the midface	number of procedures	132,128	97,780	34,348
	mean age (years, SD)	46.7 (20.2)	42.8 (18.6)	57.8 (20.6)
fracture of the lateral midface 5-760	number of procedures	102,491	75,435	27,056
	mean age (years, SD)	46.2 (20.1)	42.0 (18.3)	57.7 (20.3)
fracture of the central midface 5-761	number of procedures	944	703	241
	mean age (years, SD)	41.7 (20.2)	39.9 (18.4)	46.6 (23.2)
fracture of the central and lateral upper midface 5-762	number of procedures	14,047	10,466	3581
	mean age (years, SD)	48.5 (20.7)	44.7 (19.2)	59.5 (21.1)
combined and complex midfacial fractures 5-763	number of procedures	14,680	11,203	3477
	mean age (years, SD)	49.0 (20.5)	46.3 (19.3)	57.7 (21.7)
fracture of the mandible	number of procedures	114,468	84,695	29,773
	mean age (years, SD)	37.8 (19.5)	34.8 (17.2)	46.4 (22.8)
fracture of the mandibular body 5-764	number of procedures	66,717	50,183	16,534
	mean age (years, SD)	37.3 (19.9)	34.4 (17.5)	46.0 23.8
fracture of the mandibular ramus and condylar process 5-765	number of procedures	47,751	34,512	13,239
	mean age (years, SD)	38.5 (18.8)	35.4 (16.7)	46.8 (21.1)
fracture of the orbital wall 5-766	number of procedures	117,666	81,368	36,298
	mean age (years, SD)	47.7 (21.2)	42.0 (19.1)	59.6 (20.9)
fracture of the frontal sinus 5-767	number of procedures	9881	9054	827
	mean age (years, SD)	39.2 (18.2)	38.8 (17.8)	43.2 (21.5)

**Table 2:** Incidence of MFT-associated procedures in Germany 2005–2022. Incidence rates (95% confidence interval) per 100,000 person-years were standardized for the German population based on the Census 2011. Relative Risk (95% confidence interval).

	Total	Gender		Age Group				
		Male	Female	0–14	15–34	35–59	60–79	over 80
**All Years**	25.1 (25.0–25.2)	37.5 (37.4–37.7)	13.3 (13.3–13.4)	3.6 (3.5–3.7)	41.8 (41.6–42.0)	24.4 (24.3–24.6)	21.2 (21.0–21.4)	29.1 (28.7–29.5)
**2005–2013**	25.8 (25.0–25.2)	39.1 (38.9–39.3)	13.1 (13.0–13.2)	4.1 (4.0–4.2)	46.9 (46.5–47.2)	24.2 (24.0–24.5)	19.4 (19.2–19.6)	26.8 (26.3–27.3)
**2014–2022**	24.4 (25.0–25.2)	35.9 (35.7–36.1)	13.6 (13.5–13.7)	3.1 (3.0–3.2)	36.7 (36.4–37.0)	24.7 (24.5–24.8)	23.1 (22.8–23.3)	31.4 (30.9–31.9)
**Relative Risk**	0.95 (0.94–0.95) *	0.92 (0.91–0.92) *	1.04 (1.03–1.05) *	0.75 (0.71–0.78) *	0.78 (0.78–0.79) *	1.02 (1.01–1.03) *	1.19 (1.17–1.21) *	1.17 (1.14–1.20) *
**2005**	24.0 (23.6–24.3)	36.5 (35.9–37.1)	12.1 (11.7–12.4)	4.4 (4.0–4.7)	45.4 (44.5–46.4)	23.4 (22.9–24.0)	15.6 (15.0–16.2)	19.4 (18.0–20.8)
**2006**	25.1 (24.7–25.4)	38.5 (37.8–39.1)	12.3 (12.0–12.7)	4.3 (3.9–4.6)	47.5 (46.6–48.5)	23.6 (23.1–24.1)	17.6 (17.0–18.3)	21.0 (19.6–22.5)
**2007**	26.0 (25.6–26.3)	39.6 (39.0–40.2)	13.1 (12.7–13.4)	4.5 (4.1–4.8)	49.7 (48.7–50.7)	23.8 (23.3–24.4)	18.2 (17.5–18.8)	24.3 (22.7–25.8)
**2008**	26.2 (25.8–26.5)	40.2 (39.6–40.8)	12.9 (12.5–13.2)	4.6 (4.2–4.9)	48.9 (47.9–49.8)	24.6 (24.1–25.2)	18.1 (17.5–18.8)	26.6 (25.0–28.2)
**2009**	26.5 (26.2–26.9)	40.8 (40.1–41.4)	13.0 (12.7–13.4)	4.1 (3.7–4.3)	48.9 (47.9–49.9)	24.5 (23.9–25.1)	19.9 (19.3–20.6)	27.5 (25.9–29.1)
**2010**	25.1 (24.8–25.5)	38.0 (37.4–38.6)	13.0 (12.6–13.3)	4.4 (4.0–4.7)	45.4 (44.5–46.4)	23.2 (22.6–23.7)	19.4 (18.7–20.0)	27.4 (25.9–29.0)
**2011**	26.6 (26.3–27.0)	40.2 (39.6–40.8)	13.7 (13.3–14.0)	3.8 (3.4–4.0)	46.7 (45.7–47.7)	24.9 (24.4–25.5)	21.2 (20.5–21.9)	30.8 (29.1–32.4)
**2012**	26.9 (26.5–27.2)	40.5 (39.9–41.2)	13.9 (13.5–14.2)	3.8 (3.4–4.1)	46.7 (45.7–47.6)	25.3 (24.8–25.9)	22.1 (21.4–22.8)	30.5 (28.9–32.2)
**2013**	25.6 (25.3–26.0)	38.1 (37.5–38.7)	13.8 (13.4–14.1)	3.1 (2.7–3.3)	42.5 (41.6–43.5)	24.5 (24.0–25.1)	22.2 (21.5–22.9)	33.5 (31.8–35.2)
**2014**	24.8 (24.4–25.1)	36.8 (36.2–37.4)	13.4 (13.0–13.7)	3.4 (3.1–3.7)	40.0 (39.1–40.9)	24.3 (23.7–24.8)	22.0 (21.3–22.7)	30.2 (28.6–31.8)
**2015**	24.8 (24.4–25.1)	36.2 (35.6–36.8)	14.0 (13.7–14.4)	3.3 (3.0–3.6)	38.9 (38.0–39.8)	24.5 (23.9–25.1)	23.3 (22.6–24.0)	31.1 (29.5–32.7)
**2016**	24.7 (24.3–25.0)	36.4 (35.8–37.0)	13.7 (13.4–14.1)	2.9 (2.6–3.2)	38.8 (37.9–39.7)	24.8 (24.2–25.3)	22.5 (21.8–23.2)	31.8 (30.2–33.4)
**2017**	24.4 (24.1–24.7)	36.2 (35.7–36.8)	13.3 (12.9–13.6)	2.7 (2.4–3.0)	37.3 (36.4–38.2)	24.5 (24.0–25.1)	23.0 (22.3–23.7)	32.5 (30.9–34.0)
**2018**	25.3 (24.9–25.6)	37.6 (37.0–38.2)	13.7 (13.4–14.1)	2.9 (2.5–3.1)	39.0 (38.1–39.9)	25.1 (24.6–25.7)	24.5 (23.7–25.2)	29.9 (28.4–31.4)
**2019**	25.3 (25.0–25.6)	37.2 (36.6–37.7)	14.2 (13.8–14.6)	3.2 (2.9–3.5)	38.4 (37.5–39.3)	25.1 (24.5–25.7)	24.4 (23.7–25.1)	32.5 (31.0–34.0)
**2020**	22.6 (22.3–22.9)	32.8 (32.3–33.4)	13.0 (12.7–13.3)	3.0 (2.6–3.2)	31.0 (30.2–31.8)	23.6 (23.1–24.2)	22.3 (21.6–23.0)	31.5 (30.1–33.0)
**2021**	22.0 (21.7–22.3)	31.9 (31.3–32.4)	12.8 (12.4–13.1)	3.0 (2.7–3.2)	30.8 (30.0–31.6)	22.7 (22.1–23.2)	21.4 (20.7–22.0)	30.7 (29.3–32.1)
**2022**	25.8 (25.5–26.2)	38.1 (37.5–38.7)	14.3 (13.9–14.6)	3.2 (2.9–3.4)	36.2 (35.3–37.0)	27.3 (26.7–27.9)	24.2 (23.5–24.9)	32.3 (30.9–33.8)

* *p*-value < 0.05.

**Table 3:** Procedure-specific incidence of MFT-associated procedures in Germany 2005–2022. Incidence rates (95% confidence interval) per 100,000 person-years were standardized for the German population based on the Census 2011. Relative Risk (95% confidence interval).

	** All procedures **	** Fractures of the Midface **				
		All midfacial fractures 5-760–5-763	Lateral midface 5-760	Central midface 5-761	Central and upper lateral midface 5-762	Combined and complex fractures 5-763
**All Years**	25.1 (25.0–25.2)	8.9 (8.8–8.9)	6.9 (6.8–6.9)	1.7 (1.7–1.7)	0.9 (0.9–1.0)	1.0 (1.0–1.0)
**2005–2013**	25.8 (25.0–25.2)	9.3 (9.2–9.4)	7.5 (7.4–7.5)	1.8 (1.8–1.9)	0.8 (0.8–0.9)	0.9 (0.9–1.0)
**2014–2022**	24.4 (25.0–25.2)	8.4 (8.3–8.5)	6.3 (6.3–6.4)	1.6 (1.6–1.6)	1.0 (1.0–1.0)	1.0 (1.0–1.0)
**Relative Risk**	0.95 (0.94–0.95) *	0.90 (0.89–0.91) *	0.85 (0.84–0.86) *	0.88 (0.86–0.90) *	1.20 (1.16–1.25) *	1.08 (1.05–1.12) *
**2005**	24.0 (23.6–24.3)	8.9 (8.6–9.1)	7.2 (7.1–7.4)	1.6 (1.5–1.7)	0.8 (0.7–0.8)	0.8 (0.7–0.9)
**2006**	25.1 (24.7–25.4)	9.0 (8.8–9.2)	7.3 (7.2–7.5)	1.8 (1.7–1.9)	0.9 (0.8–0.9)	0.8 (0.7–0.8)
**2007**	26.0 (25.6–26.3)	9.3 (9.1–9.5)	7.6 (7.4–7.8)	1.8 (1.7–1.9)	0.8 (0.7–0.8)	0.8 (0.7–0.9)
**2008**	26.2 (25.8–26.5)	9.4 (9.2–9.7)	7.7 (7.5–7.9)	2.0 (1.9–2.1)	0.7 (0.7–0.8)	0.9 (0.8–1.0)
**2009**	26.5 (26.2–26.9)	9.7 (9.5–9.9)	7.8 (7.6–8.0)	2.1 (2.0–2.2)	0.8 (0.7–0.9)	1.0 (0.9–1.1)
**2010**	25.1 (24.8–25.5)	9.1 (8.9–9.3)	7.2 (7.0–7.4)	1.7 (1.6–1.7)	0.8 (0.7–0.8)	1.0 (1.0–1.1)
**2011**	26.6 (26.3–27.0)	9.6 (9.3–9.8)	7.5 (7.3–7.6)	2.0 (1.9–2.1)	1.0 (0.9–1.0)	1.1 (1.0–1.1)
**2012**	26.9 (26.5–27.2)	9.6 (9.4–9.8)	7.6 (7.4–7.8)	1.8 (1.7–1.9)	1.0 (0.9–1.1)	1.0 (0.9–1.1)
**2013**	25.6 (25.3–26.0)	9.3 (9.0–9.5)	7.1 (6.9–7.3)	1.7 (1.6–1.8)	1.0 (0.9–1.0)	1.1 (1.0–1.2)
**2014**	24.8 (24.4–25.1)	8.8 (8.5–9.0)	6.9 (6.7–7.1)	1.7 (1.6–1.8)	0.9 (0.8–1.0)	0.9 (0.9–1.0)
**2015**	24.8 (24.4–25.1)	8.7 (8.5–8.9)	6.7 (6.5–6.9)	1.7 (1.6–1.8)	0.9 (0.9–1.0)	1.0 (1.0–1.1)
**2016**	24.7 (24.3–25.0)	8.8 (8.6–9.0)	6.6 (6.4–6.8)	1.7 (1.6–1.8)	1.0 (0.9–1.1)	1.1 (1.0–1.2)
**2017**	24.4 (24.1–24.7)	8.4 (8.2–8.6)	6.4 (6.2–6.5)	1.7 (1.6–1.7)	1.0 (1.0–1.1)	1.0 (0.9–1.0)
**2018**	25.3 (24.9–25.6)	8.6 (8.4–8.8)	6.4 (6.3–6.6)	1.7 (1.6–1.8)	1.1 (1.0–1.2)	1.0 (0.9–1.1)
**2019**	25.3 (25.0–25.6)	8.5 (8.3–8.7)	6.3 (6.1–6.4)	1.5 (1.4–1.6)	1.1 (1.1–1.2)	1.0 (1.0–1.1)
**2020**	22.6 (22.3–22.9)	7.7 (7.5–7.9)	5.6 (5.5–5.8)	1.5 (1.4–1.6)	1.0 (0.9–1.0)	1.0 (1.0–1.1)
**2021**	22.0 (21.7–22.3)	7.4 (7.2–7.6)	5.5 (5.3–5.7)	1.5 (1.4–1.5)	1.0 (0.9–1.0)	0.9 (0.9–1.0)
**2022**	25.8 (25.5–26.2)	8.8 (8.6–9.0)	6.4 (6.2–6.6)	1.6 (1.5–1.7)	1.2 (1.1–1.3)	1.1 (1.0–1.2)
	**All procedures**	** Fractures of the Mandible **			**Fractures of the orbital wall** 5-766	**Fractures of the frontal sinus** 5-767
		All mandibular fractures 5-764–5-765	Mandibular body 5-764	Mandibular ramus And condylar process 5-765		
**All Years**	4.2 (4.1–4.3)	4.5 (4.4–4.5)	3.2 (3.2–3.2)	7.9 (7.8–7.9)	0.7 (0.6–0.7)	4.2 (4.1–4.3)
**2005–2013**	4.3 (4.2–4.5)	4.6 (4.6–4.7)	3.1 (3.1–3.2)	8.0 (7.9–8.0)	0.7 (0.7–0.7)	4.3 (4.2–4.5)
**2014–2022**	4.0 (3.9–4.1)	4.3 (4.3–4.4)	3.3 (3.2–3.3)	7.8 (7.7–7.8)	0.6 (0.6–0.6)	4.0 (3.9–4.1)
**Relative Risk**	0.92 (0.88–0.96) *	0.93 (0.91–0.94) *	1.04 (1.03–1.06) *	0.98 (0.97–0.99) *	0.86 (0.83–0.90) *	0.92 (0.88–0.96) *
**2005**	7.7 (7.5–7.8)	4.9 (4.7–5.0)	2.8 (2.6–2.9)	6.8 (6.7–7.0)	0.6 (0.6–0.7)	7.7 (7.5–7.8)
**2006**	7.9 (7.7–8.1)	4.9 (4.8–5.1)	3.0 (2.9–3.2)	7.4 (7.2–7.6)	0.7 (0.6–0.7)	7.9 (7.7–8.1)
**2007**	8.2 (8.0–8.4)	4.7 (4.6–4.9)	3.4 (3.3–3.5)	7.8 (7.6–8.0)	0.7 (0.6–0.8)	8.2 (8.0–8.4)
**2008**	8.0 (7.8–8.2)	4.7 (4.6–4.9)	3.2 (3.1–3.4)	8.1 (7.9–8.3)	0.7 (0.7–0.8)	8.0 (7.8–8.2)
**2009**	7.9 (7.7–8.0)	4.7 (4.5–4.8)	3.2 (3.1–3.3)	8.2 (8.0–8.4)	0.7 (0.7–0.8)	7.9 (7.7–8.0)
**2010**	7.5 (7.3–7.7)	4.4 (4.3–4.6)	3.0 (2.9–3.2)	7.8 (7.6–7.9)	0.8 (0.7–0.8)	7.5 (7.3–7.7)
**2011**	7.8 (7.6–8.0)	4.7 (4.6–4.9)	3.1 (3.0–3.2)	8.5 (8.3–8.7)	0.7 (0.7–0.8)	7.8 (7.6–8.0)
**2012**	7.7 (7.5–7.9)	4.4 (4.3–4.6)	3.3 (3.2–3.4)	8.8 (8.6–9.0)	0.8 (0.7–0.8)	7.7 (7.5–7.9)
**2013**	7.5 (7.4–7.7)	4.3 (4.1–4.4)	3.3 (3.1–3.4)	8.1 (7.9–8.3)	0.7 (0.6–0.8)	7.5 (7.4–7.7)
**2014**	7.5 (7.3–7.7)	4.2 (4.1–4.4)	3.2 (3.1–3.4)	7.8 (7.7–8.0)	0.7 (0.6–0.7)	7.5 (7.3–7.7)
**2015**	7.3 (7.1–7.5)	3.9 (3.8–4.0)	3.4 (3.2–3.5)	8.1 (7.9–8.3)	0.7 (0.6–0.7)	7.3 (7.1–7.5)
**2016**	7.4 (7.2–7.6)	4.1 (4.0–4.3)	3.3 (3.2–3.4)	7.9 (7.7–8.1)	0.6 (0.5–0.6)	7.4 (7.2–7.6)
**2017**	7.5 (7.3–7.7)	4.5 (4.3–4.6)	3.0 (2.9–3.2)	7.9 (7.7–8.0)	0.6 (0.6–0.7)	7.5 (7.3–7.7)
**2018**	7.9 (7.7–8.0)	4.6 (4.4–4.7)	3.3 (3.1–3.4)	8.2 (8.0–8.4)	0.7 (0.6–0.7)	7.9 (7.7–8.0)
**2019**	8.0 (7.8–8.2)	4.6 (4.5–4.8)	3.4 (3.3–3.5)	8.1 (7.9–8.3)	0.6 (0.6–0.7)	8.0 (7.8–8.2)
**2020**	7.2 (7.0–7.4)	4.1 (4.0–4.3)	3.1 (3.0–3.2)	7.1 (7.0–7.3)	0.5 (0.5–0.6)	7.2 (7.0–7.4)
**2021**	7.2 (7.0–7.3)	4.0 (3.8–4.1)	3.2 (3.1–3.3)	6.9 (6.7–7.0)	0.6 (0.5–0.6)	7.2 (7.0–7.3)
**2022**	8.4 (8.2–8.6)	4.6 (4.5–4.8)	3.7 (3.6–3.9)	8.1 (7.9–8.3)	0.6 (0.5–0.6)	8.4 (8.2–8.6)

* *p*-value < 0.05.

**Table 4:** Regional incidence of MFT = associated procedures in all 16 federal states of Germany 2005–2022. Incidence rates (95% confidence interval) per 100,000 person-years standardized to the German population based on the Census 2011. Relative Risk (95% confidence interval).

	**Baden-Wuerttemberg**	**Bavaria**	**Berlin**	**Brandenburg**	**Bremen**	**Hamburg**	**Hesse**	**Mecklenburg-Vorpommern**
**All Years**	23.5 (23.2–23.7)	23.6 (23.4–23.8)	30.6 (30.2–31.0)	21.5 (21.1–21.9)	57.5 (56.1–58.9)	54.0 (53.2–54.8)	25.1 (24.8–25.4)	36.4 (35.7–37.1)
**2005–2013**	24.1 (23.8–24.4)	23.9 (23.6–24.2)	30.0 (29.4–30.6)	23.4 (22.7–24.0)	60.4 (58.4–62.3)	52.1 (51.0–53.2)	25.3 (24.8–25.7)	43.0 (42.0–44.1)
**2014–2022**	22.8 (22.5–23.1)	23.2 (22.9–23.5)	31.2 (30.6–31.8)	19.7 (19.1–20.2)	54.6 (52.8–56.5)	55.9 (54.7–57.0)	25.0 (24.6–25.4)	29.8 (28.9–30.7)
**RR**	0.94 (0.93–0.96) *	0.97 (0.95–0.99) *	1.04 (1.01–1.07) *	0.84 (0.81–0.88) *	0.91 (0.86–0.95) *	1.07 (1.04–1.10) *	0.99 (0.97–1.01)	0.69 (0.67–0.72) *
**2005**	23.3 (22.4–24.2)	22.4 (21.6–23.2)	30.8 (28.9–32.6)	21.7 (19.9–23.5)	59.0 (53.2–64.9)	45.5 (42.3–48.7)	19.8 (18.7–20.9)	42.1 (39.0–45.2)
**2006**	23.5 (22.6–24.5)	21.6 (20.8–22.5)	33.5 (31.5–35.4)	22.2 (20.4–24.0)	61.3 (55.3–67.2)	50.4 (47.1–53.8)	18.9 (17.8–20.0)	45.7 (42.5–48.9)
**2007**	24.7 (23.8–25.6)	23.7 (22.8–24.5)	31.0 (29.1–32.8)	21.9 (20.1–23.7)	63.1 (57.0–69.1)	51.5 (48.1–54.8)	21.4 (20.3–22.6)	48.5 (45.2–51.9)
**2008**	24.1 (23.2–25.0)	23.6 (22.8–24.5)	30.3 (28.5–32.2)	24.5 (22.6–26.5)	70.4 (64.0–76.8)	52.2 (48.9–55.6)	24.7 (23.5–26.0)	46.0 (42.8–49.3)
**2009**	23.8 (22.9–24.7)	25.1 (24.3–26.0)	30.9 (29.0–32.7)	24.8 (22.9–26.8)	66.1 (59.9–72.3)	53.6 (50.2–57.0)	27.6 (26.3–28.9)	45.0 (41.8–48.2)
**2010**	24.6 (23.6–25.5)	24.5 (23.6–25.4)	26.1 (24.4–27.8)	21.6 (19.8–23.4)	62.5 (56.4–68.5)	47.0 (43.8–50.2)	27.8 (26.4–29.1)	42.5 (39.4–45.7)
**2011**	25.3 (24.3–26.2)	24.2 (23.3–25.1)	27.3 (25.5–29.1)	23.6 (21.7–25.5)	57.6 (51.8–63.4)	48.6 (45.3–51.9)	30.1 (28.8–31.5)	38.8 (35.7–41.8)
**2012**	24.1 (23.1–25.0)	24.8 (23.9–25.6)	29.5 (27.6–31.3)	26.9 (24.9–29.0)	59.0 (53.1–64.9)	60.1 (56.5–63.8)	29.4 (28.1–30.8)	40.0 (36.9–43.1)
**2013**	23.8 (22.9–24.8)	25.4 (24.5–26.2)	30.4 (28.6–32.3)	23.0 (21.1–24.9)	44.2 (39.1–49.3)	59.9 (56.3–63.6)	27.6 (26.2–28.9)	38.6 (35.5–41.6)
**2014**	23.0 (22.1–23.9)	22.9 (22.1–23.8)	30.6 (28.7–32.4)	22.5 (20.7–24.4)	58.5 (52.7–64.3)	58.0 (54.5–61.6)	25.6 (24.3–26.8)	35.1 (32.2–38.0)
**2015**	22.9 (22.0–23.8)	23.8 (23.0–24.7)	26.1 (24.4–27.8)	18.8 (17.1–20.5)	59.8 (54.0–65.7)	60.4 (56.8–64.0)	25.1 (23.8–26.3)	31.1 (28.3–33.8)
**2016**	23.1 (22.2–24.0)	25.2 (24.4–26.1)	28.1 (26.4–29.8)	18.9 (17.2–20.6)	58.3 (52.6–64.1)	62.0 (58.4–65.6)	26.2 (24.9–27.4)	29.7 (27.1–32.4)
**2017**	21.6 (20.8–22.5)	24.2 (23.4–25.0)	28.2 (26.4–29.9)	20.3 (18.5–22.0)	59.2 (53.4–65.0)	58.7 (55.2–62.2)	25.7 (24.5–27.0)	28.1 (25.5–30.7)
**2018**	23.3 (22.4–24.1)	24.5 (23.7–25.4)	31.3 (29.4–33.1)	21.4 (19.6–23.2)	50.6 (45.3–55.9)	52.6 (49.3–56.0)	25.5 (24.3–26.8)	28.4 (25.8–31.0)
**2019**	23.2 (22.3–24.1)	24.0 (23.2–24.9)	34.5 (32.6–36.4)	20.5 (18.7–22.2)	55.1 (49.6–60.7)	56.9 (53.4–60.3)	26.0 (24.8–27.3)	28.8 (26.2–31.4)
**2020**	23.5 (22.6–24.4)	21.4 (20.6–22.2)	33.3 (31.5–35.2)	18.9 (17.2–20.6)	56.4 (50.8–62.1)	52.9 (49.6–56.2)	23.8 (22.6–25.0)	24.8 (22.4–27.2)
**2021**	21.0 (20.2–21.9)	19.7 (18.9–20.4)	31.1 (29.3–32.9)	16.5 (15.0–18.1)	48.5 (43.3–53.8)	49.5 (46.3–52.7)	22.8 (21.6–24.0)	28.9 (26.2–31.5)
**2022**	23.5 (22.6–24.3)	22.8 (21.9–23.6)	37.7 (35.7–39.6)	19.3 (17.6–21.0)	45.2 (40.1–50.2)	51.9 (48.7–55.2)	24.4 (23.1–25.6)	33.5 (30.7–36.3)
	**Lower Saxony**	**North Rhine-Westphalia**	**Rhineland-** **Palatinate**	**Saarland**	**Saxony**	**Saxony-** **Anhalt**	**Schleswig-Holstein**	**Thuringia**
**All Years**	25.5 (25.2–25.8)	27.2 (27.0–27.4)	20.3 (20.0–20.6)	34.6 (33.7–35.5)	31.0 (30.6–31.4)	28.3 (27.8–28.8)	22.6 (22.2–23.0)	34.8 (34.2–35.4)
**2005–2013**	27.9 (27.5–28.3)	28.3 (28.0–28.6)	18.7 (18.2–19.1)	37.3 (36.0–38.5)	28.9 (28.4–29.5)	30.0 (29.3–30.8)	24.6 (24.0–25.3)	32.1 (31.3–32.9)
**2014–2022**	23.1 (22.7–23.4)	26.1 (25.8–26.3)	21.9 (21.5–22.4)	32.0 (30.8–33.1)	33.1 (32.5–33.6)	26.6 (25.9–27.3)	20.5 (20.0–21.1)	37.5 (36.6–38.3)
**RR**	0.83 (0.81–0.85) *	0.92 (0.91–0.93) *	1.17 (1.14–1.21) *	0.86 (0.82–0.90) *	1.14 (1.11–1.17) *	0.88 (0.85–0.92) *	0.83 (0.80–0.86) *	1.17 (1.13–1.21) *
**2005**	28.6 (27.4–29.7)	26.3 (25.6–27.1)	16.3 (15.0–17.5)	41.8 (37.9–45.7)	29.2 (27.5–30.8)	28.9 (26.8–31.1)	22.8 (21.1–24.6)	29.3 (27.1–31.4)
**2006**	27.4 (26.3–28.6)	27.2 (26.5–28.0)	14.7 (13.5–15.9)	36.3 (32.6–39.9)	29.7 (28.0–31.3)	27.2 (25.1–29.3)	24.8 (23.0–26.7)	27.8 (25.7–30.0)
**2007**	28.0 (26.9–29.2)	27.9 (27.2–28.7)	17.6 (16.3–18.9)	41.5 (37.6–45.4)	31.1 (29.4–32.7)	29.8 (27.6–32.0)	23.2 (21.5–25.0)	30.0 (27.8–32.3)
**2008**	27.7 (26.6–28.9)	28.6 (27.9–29.4)	19.6 (18.2–21.0)	39.9 (36.1–43.8)	29.9 (28.3–31.6)	30.6 (28.4–32.8)	27.9 (26.0–29.8)	29.8 (27.6–32.1)
**2009**	29.9 (28.7–31.1)	28.9 (28.1–29.7)	19.9 (18.5–21.2)	35.3 (31.7–39.0)	27.9 (26.3–29.5)	31.4 (29.1–33.6)	25.9 (24.0–27.8)	31.9 (29.5–34.2)
**2010**	27.6 (26.4–28.7)	28.2 (27.4–29.0)	17.1 (15.8–18.4)	39.7 (35.8–43.6)	25.9 (24.4–27.5)	29.4 (27.2–31.6)	23.2 (21.5–25.0)	33.2 (30.8–35.6)
**2011**	27.1 (26.0–28.3)	29.4 (28.6–30.2)	19.9 (18.5–21.3)	33.4 (29.8–36.9)	28.1 (26.5–29.7)	31.6 (29.3–33.9)	26.2 (24.3–28.1)	34.5 (32.0–36.9)
**2012**	27.1 (26.0–28.3)	29.3 (28.5–30.1)	22.7 (21.2–24.2)	35.5 (31.8–39.2)	28.7 (27.0–30.3)	30.1 (27.9–32.4)	25.1 (23.3–27.0)	35.2 (32.7–37.7)
**2013**	27.4 (26.3–28.6)	28.8 (28.0–29.6)	20.4 (19.0–21.8)	31.8 (28.3–35.4)	30.0 (28.3–31.7)	31.3 (29.0–33.6)	22.5 (20.7–24.2)	37.0 (34.5–39.6)
**2014**	23.1 (22.1–24.2)	27.2 (26.4–27.9)	20.8 (19.4–22.2)	34.3 (30.6–37.9)	31.5 (29.7–33.2)	29.6 (27.4–31.9)	21.9 (20.2–23.6)	39.4 (36.7–42.0)
**2015**	22.0 (21.0–23.0)	26.0 (25.3–26.8)	19.9 (18.5–21.2)	35.2 (31.5–38.8)	33.8 (32.0–35.6)	28.2 (26.0–30.4)	23.6 (21.8–25.4)	37.3 (34.8–39.9)
**2016**	23.9 (22.8–25.0)	26.2 (25.5–27.0)	19.8 (18.5–21.2)	34.7 (31.0–38.3)	31.8 (30.1–33.5)	29.6 (27.3–31.8)	20.4 (18.7–22.0)	39.0 (36.4–41.6)
**2017**	24.2 (23.1–25.2)	26.3 (25.5–27.0)	21.7 (20.2–23.1)	30.6 (27.2–34.1)	31.3 (29.6–33.0)	27.0 (24.9–29.2)	20.7 (19.1–22.4)	38.6 (36.0–41.2)
**2018**	24.9 (23.8–26.0)	26.9 (26.2–27.7)	22.3 (20.9–23.8)	34.2 (30.5–37.8)	32.5 (30.8–34.3)	27.1 (24.9–29.3)	20.5 (18.9–22.2)	38.1 (35.4–40.7)
**2019**	24.8 (23.7–25.9)	26.4 (25.7–27.2)	22.8 (21.4–24.3)	35.5 (31.8–39.3)	35.0 (33.2–36.8)	26.8 (24.6–28.9)	19.8 (18.1–21.4)	41.1 (38.4–43.8)
**2020**	21.5 (20.5–22.5)	24.8 (24.0–25.5)	23.1 (21.6–24.5)	28.7 (25.3–32.0)	35.2 (33.3–37.0)	24.3 (22.2–26.4)	18.9 (17.4–20.5)	36.2 (33.6–38.7)
**2021**	20.5 (19.5–21.5)	24.4 (23.7–25.2)	22.4 (21.0–23.9)	26.1 (22.9–29.3)	31.4 (29.7–33.2)	22.0 (20.0–24.0)	17.6 (16.1–19.2)	35.0 (32.4–37.5)
**2022**	22.9 (21.9–24.0)	26.2 (25.5–27.0)	24.7 (23.2–26.2)	28.4 (25.1–31.7)	35.0 (33.2–36.8)	24.5 (22.4–26.6)	21.2 (19.6–22.9)	32.7 (30.3–35.2)

* *p*-value < 0.05.

**Table 5:** Results of the Poisson regression models for the total population and stratified by gender, fracture localization and age group. (Germany, 2005–2022). Relative risk for MFT-associated procedure (95% Confidence interval).

Population	Total	Age Group				
All		0–14	15–34	35–59	60–79	Over 80
Calendar year	0.995 (0.994–0.996) *	0.975 (0.970–0.979) *	0.976 (0.975–0.977) *	1.002 (1.001–1.003) *	1.019 (1.017–1.020) *	1.019 (1.016–1.021) *
Gender ^a^	2.819 (2.799–2.840) *	1.689 (1.610–1.772) *	6.149 (6.057–6.243) *	2.852 (2.816–2.887) *	1.454 (1.432–1.476) *	0.989 (0.963–1.015)
Age Group		0.147 (0.143–0.150) *	1.712* (1.700–1.725) *	1.000 ^b^	0.905 (0.897–0.913) *	1.425 (1.406–1.445) *
**Males**						
Calendar year	0.991 (0.990–0.992) *	0.977 (0.972–0.982) *	0.974 (0.973–0.975) *	1.001 (1.000–1.002) *	1.018 (1.016–1.020) *	1.024 (1.020–1.028) *
Age Group		0.125 (0.122–0.129) *	1.978 * (1.961–1.994) *	1.000 ^b^	0.713 (0.704–0.721) *	0.833 (0.815–0.851) *
**Females**						
Calendar year	1.007 (1.005–1.008) *	0.970 (0.963–0.977) *	0.988 (0.985–0.990) *	1.005 (1.003–1.007) *	1.020 (1.017–1.022) *	1.016 (1.013–1.019) *
Age Group		0.211 (0.203–0.220) *	0.917 * (0.901–0.934) *	1.000 ^b^	1.390 (1.368–1.411) *	2.326 (2.283–2.370) *
**Fractures of the Midface**						
Calendar year	0.990 (0.989–0.991) *	0.920 (0.910–0.931) *	0.969 (0.967–0.971) *	0.992 (0.991–0.994) *	1.016 (1.014–1.018) *	1.013 (1.009–1.016) *
Gender ^a^	3.015 (2.978–3.053) *	2.137 (1.894–2.411) *	7.711 (7.484–7.945) *	2.998 (2.939–3.060) *	1.711 (1.669–1.754) *	1.182 (1.136–1.230) *
Age Group		0.064 (0.061–0.068) *	1.341* (1.324–1.358) *	1.000 ^b^	0.903 (0.890–0.917) *	1.508 (1.476–1.540) *
**Fractures of the mandible**						
Calendar year	0.996 (0.995–0.998) *	0.967 (0.962–0.973) *	0.983 (0.981–0.985) *	1.011 (1.009–1.013) *	1.020 (1.016–1.023) *	1.016 (1.010–1.022) *
Gender ^a^	2.806 (2.770–2.844) *	1.601 (1.501–1.708) *	4.931 (4.822–5.042) *	2.348 (2.296–2.402) *	1.420 (1.373–1.469) *	0.784 (0.735–0.837) *
Age Group		0.289 (0.280–0.299) *	2.453* (2.421–2.486) *	1.000 ^b^	0.660 (0.647–0.673) *	0.899 (0.871–0.928) *
**Fractures of the orbital wall**						
Calendar year	0.999 (0.998–1.000)	0.949 (0.941–0.956) *	0.977 (0.975–0.978) *	1.002 (1.000–1.004) *	1.022 (1.022–1.024) *	1.028 (1.024–1.032) *
Gender ^a^	2.379 (2.350–2.409) *	1.674 (1.536–1.824) *	6.632 (6.435–6.836) *	2.735 (2.675–2.796) *	1.151 (1.123–1.179) *	0.845 (0.810–0.881) *
Age Group		0.148 (0.142–0.154) *	1.487 * (1.466–1.508) *	1.000 ^b^	1.147 (1.129–1.165) *	1.842 (1.803–1.883) *
**Fractures of the frontal sinus**						
Calendar year	0.999 (0.998–1.001)	0.937 (0.929–0.945) *	0.997 (0.995–0.999) *	0.998 (0.996–1.000)	1.022 (1.019–1.026) *	1.015 (1.007–1.023) *
Gender ^a^	11.982 (11.690–12.281) *	3.106 (2.807–3.437) *	17.632 (16.865–18.434) *	12.177 (11.676–12.699) *	8.531 (8.069–9.020) *	6.308 (5.738–6.934) *
Age Group	0.995 (0.994–0.996) *	0.975 (0.970–0.979) *	1.952 (1.924–1.981) *	1.000 ^b^	0.683 (0.669–0.697) *	0.660 (0.633–0.687) *

* *p*-value < 0.05; ^a^ Baseline: gender: female, age: 35–59 years; ^b^ Baseline: age: 35–59 years.

**Table 6:** Results of the Poisson models stratified by federal state (Germany, 2005–2022). Relative risk for MFT-associated procedure (95% Confidence interval).

	**Federal State**							
	**Baden-Wuerttemberg**	**Bavaria**	**Berlin**	**Brandenburg**	**Bremen**	**Hamburg**	**Hesse**	**Mecklenburg-Vorpommern**
**Risk factor**								
**Calendar year**	0.995 (0.993–0.996) *	0.997 (0.995–0.999) *	1.001 (0.998–1.004)	0.982 (0.978–0.986) *	0.989 (0.985–0.993) *	1.004 (1.002–1.007) *	1.005 (1.002–1.007) *	0.964 (0.960–0.967) *
**Gender ^a^**	2.949 (2.888–3.011) *	2.730 (2.679–2.782) *	2.969 (2.877–3.065) *	2.715 (2.593–2.843) *	2.171 (2.079–2.267) *	2.398 (2.323–2.476) *	2.857 (2.781–2.934) *	2.938 (2.816–3.065) *
**Age Group**								
0–14	0.223 (0.211–0.236) *	0.185 (0.175–0.195) *	0.112 (0.100- 0.125) *	0.133 (0.113–0.157) *	0.161 (0.144–0.180) *	0.136 (0.120–0.154) *	0.179 (0.166–0.194) *	0.288 (0.258–0.321) *
15–34	1.721 (1.685–1.758) *	1.661 (1.628–1.694) *	1.511 (1.465–1.560) *	1.945 (1.854–2.039) *	1.440 (1.365–1.520) *	1.498 (1.445–1.554) *	1.694 (1.648–1.741) *	1.960 (1.878–2.046) *
35–59	1.000 ^a^	1.000 ^a^	1.000 ^a^	1.000 ^a^	1.000 ^a^	1.000 ^a^	1.000 ^a^	1.000 ^a^
60–79	0.977 (0.951–1.003)	0.968 (0.945–0.992) *	0.809 (0.775–0.844) *	0.802 (0.757–0.850) *	0.770 (0.723–0.820) *	0966 (0.927–1.007)	0.914 (0.883–0.945) *	0.718 (0.678–0.759) *
80+	1.371 (1.317–1.427) *	1.367 (1.318–1.419) *	0.975 (0.916–1.038)	1.008 (0.930–1.092)	0.904 (0.851–0.960) *	1.508 (1.442–1.577) *	1.383 (1.315–1.456) *	0.805 (0.751–0.863) *
	**Lower Saxony**	**North Rhine-Westphalia**	**Rhineland-** **Palatinate**	**Saarland**	**Saxony**	**Saxony-** **Anhalt**	**Schleswig-Holstein**	**Thuringia**
**Risk factor**								
**Calendar year**	0.983 (0.981–0.985) *	0.994 (0.992–0.995) *	1.023 (1.020–1.026) *	0.981 (0.977–0.986) *	1.009 (1.007–1.012) *	0.983 (0.873–1.008)	0.987 (0.983–0.991) *	1.012 (1.009–1.016) *
**Gender ^a^**	2.774 (2.710–2.839) *	2.749 (2.709–2.790) *	2.851 (2.749–2.957) *	2.716 (2.577–2.862) *	2.799 (2.718–2.882) *	2.829 (2.713–2.949) *	2.628 (2.521–2.740) *	2.644 (2.549–2.743) *
**Age Group**								
0–14	0.136 (0.126–0.147) *	0.167 (0.159–0.175) *	0.183 (0.165–0.203) *	0.189 (0.164–0.217) *	0.143 (0.130–0.158) *	0.157 (0.134–0.185) *	0.195 (0.173–0.219) *	0.158 (0.136–0.183) *
15–34	1.688 (1.648–1.729) *	1.735 (1.708–1.762) *	1.829 (1.763–1.898) *	1.963 (1.852–2.081) *	1.651 (1.601–1.702) *	1.953 (1.871–2.037) *	1.751 (1.674–1.831) *	1.859 (1.786–1.936) *
35–59	1.000 ^a^	1.000 ^a^	1.000 ^a^	1.000 ^a^	1.000 ^a^	1.000 ^a^	1.000 ^a^	1.000 ^a^
60–79	0.814 (0.790–0.839) *	0.937 (0.919–0.955) *	0.864 (0.824–0.906) *	0.828 (0.767–0.893) *	0.803 (0.775–0.833) *	0.720 (0.683–0.759) *	0.957 (0.908–1.009)	1.006 (0.962–1.053) *
80+	0.983 (0.981–0.985) *	0.994 (0.992–0.995) *	1.023 (1.020–1.026) *	0.981 (0.977–0.986) *	1.009 (1.007–1.012) *	0.983 (0.873–1.008)	0.987 (0.983–0.991) *	1.012 (1.009–1.016) *

* *p*-value < 0.05; ^a^ Baseline: gender: female, age: 35–59 years.

## Data Availability

Publicly available anonymized claims data were provided by the German Federal Statistical Office (Statistisches Bundesamt—Destatis. Genesis-Online. Data license by-2-0.
